# Anti‐oral cancer effects of triptolide by downregulation of DcR3 in vitro, in vivo, and in preclinical patient‐derived tumor xenograft model

**DOI:** 10.1002/hed.25554

**Published:** 2018-12-10

**Authors:** Cheng‐Yu Yang, Chih‐Kung Lin, Cheng‐Chih Hsieh, Chang‐Huei Tsao, Chun‐Shu Lin, Bo Peng, Yen‐Tzu Chen, Chun‐Chieh Ting, Wei‐Chin Chang, Gu‐Jiun Lin, Huey‐Kang Sytwu, Yuan‐Wu Chen

**Affiliations:** ^1^ School of Dentistry, National Defense Medical Center Taipei Taiwan; ^2^ Division of Anatomic Pathology Taipei Tzu Chi Hospital Taipei Taiwan; ^3^ Department of Pharmacy Practice Tri‐Service General Hospital Taipei Taiwan; ^4^ Graduate Institute of Microbiology and Immunology National Defense Medical Center Taipei Taiwan; ^5^ Department of Medical Research Tri‐Service General Hospital Taipei Taiwan; ^6^ Department of Radiation Oncology Tri‐Service General Hospital, National Defense Medical Centre Taipei Taiwan; ^7^ Graduate Institute of Clinical Medicine, College of Medicine, Taipei Medical University Taipei Taiwan; ^8^ Department of Oral and Maxillofacial Surgery Tri‐Service General Hospital Taipei Taiwan; ^9^ Department of Biology and Anatomy National Defense Medical Center Taipei Taiwan

**Keywords:** decoy receptor 3 (DcR3), metastasis‐associated protein 1 (MTA1), oral squamous cell carcinoma (OSCC), patient‐derived tumor xenograft (PDTX), triptolide

## Abstract

**Background:**

Aberrant expression of decoy receptor 3 (DcR3) is considered to be a diagnostic and therapeutic target for human cancers. The aim of this study was to assess DcR3 as a target of the anticancer effects of triptolide (TPL) in preclinical patient‐derived tumor xenograft (PDTX) models of oral squamous cell carcinoma (OSCC).

**Methods:**

The expression of DcR3 was evaluated through immunohistochemistry, and correlations were examined using clinical variables. The effects of TPL on the expression of DcR3 and cell proliferation were investigated in OSCC cell lines and in PDTX models.

**Results:**

DcR3 overexpression was associated with overall survival and tumor size. TPL significantly decreased tumor growth. Moreover, TPL inhibited the expression of metastasis‐associated protein 1 (MTA1), a transcription factor for DcR3 in vivo, in vitro, and in PDTX models.

**Conclusion:**

TPL appeared to exert anticancer effects by repressing DcR3 and MTA1 in vitro, in vivo, and in PDTX models.

## 1 INTRODUCTION

Oral squamous cell carcinoma (OSCC) is the most common malignant tumor of the head and neck; it is the eighth most prevalent malignancy worldwide and the third most common cancer in developing countries.^1^ Furthermore, oral cancer causes disfigurement and disability, and has a painful prognosis.^2^, ^3^ Concurrent chemoradiotherapy has demonstrated efficacy in organ preservation but limited improvement in survival rates in patients with head and neck cancer. Therefore, the discovery of potential therapeutic drugs for treating advanced oral cancer is crucial.

Decoy receptor 3 (DcR3 or TNFRSF6B) is a soluble receptor belonging to the tumor necrosis factor receptor superfamily (TNFRSF) that binds competitively to other TNFSF members, such as Fas ligand (FasL/TNFSF6/CD95L),^4^ LIGHT (TNFSF14),^5^ and TNF‐like molecule 1A (TL1A/TNFSF15).^6^ Upregulation of DcR3 was associated with poor prognosis in several malignancies^7^, ^8^, ^9^, ^10^, ^11^, ^12^, ^13^, ^14^, ^15^, ^16^ due to its effect on angiogenesis and the proliferation, invasion, and metastasis of tumor cells.^7^, ^8^, ^9^, ^17^, ^18^, ^19^ However, very few studies have explored the clinicopathological role of DcR3 in oral cancer. Epidermal growth factor receptor (EGFR) is overexpressed in OSCC and is associated with poor prognosis.^20^, ^21^, ^22^ Activation of EGFR by epidermal growth factor and transforming growth factor‐alpha markedly upregulates DcR3 production in keratinocytes.^23^ MTA1 expression in immortalized keratinocytes has been shown to partially depend on the activation of the EGFR.^24^ Notably, studies that have applied data mining to analyze DcR3 promoter using bioinformatic tools on the GENECARD web site (www.genecard.com) have revealed that MTA1 is a transcription factor of DcR3. Overexpression of *MTA1* is associated with the progression of various cancer types, including those of the head and neck.^25^, ^26^ These results suggest that the correlation between DcR3 and MTA1 might contribute to cancer progression in patients with OSCC.

Appropriate preclinical models could advance cancer drug research. A patient‐derived tumor xenograft (PDTX) model has numerous advantages over standard xenograft models in preclinical trials of novel anticancer drugs because PDTXs are more capable of retaining the genetic, molecular, and histological heterogeneity of patient tumors through serial passage in a mouse model.^27^, ^28^, ^29^

Herbal extracts and phytochemicals have recently been assessed for their inhibitory ability against cancer cell growth and metastasis.^30^, ^31^ These compounds are suggested as candidates for novel chemotherapeutic agents or adjuvants that improve anticancer effects in combination with standard treatments. Triptolide (TPL, C_20_H_24_O_6_), a diterpenoid triepoxide derived from the Chinese herb *Tripterygium wilfordii*, exerts effects against cancer,^31^, ^32^, ^33^ including oral cancer.^34^, ^35^, ^36^ These findings have indicated that TPL might be a promising candidate for combined therapy for advanced oral cancer. TPL can suppress EGFR levels in vitro and in vivo in malignant tumors.^37^, ^38^ TPL can also downregulate the expression of DcR3 in pancreatic cancer cells.^39^ However, the advanced anticancer mechanisms of TPL in OSCC remain unexplored.

In the present study, we assessed the expression of DcR3 in oral cancer cells using human tumor tissue arrays. We evaluated the anticancer effects of TPL through the downregulation of DcR3 in our PDTX models in vivo and in vitro.

## 2 MATERIALS AND METHODS

### 2.1 Human tissue microarray

Microarray slides were prepared using tissues from paraffin‐embedded primary OSCC tumors (from 99 patients) and normal oral mucosa (from 10 patients). Tissue samples were extracted from a representative area of each paraffin‐embedded tumor block. The methods used were as described in our previous study.^40^ The microarray study was approved by the Ethics Review Committee of the Tri‐Service General Hospital, Taipei, Taiwan (IRB: TSGH‐1‐101‐05‐092).

### 2.2 Establishment of PDTX models and treatment protocol

The methods for establishing a PDTX were described in our previous study.^40^ Briefly, tumor specimens were obtained from patients with OSCC during the initial surgical treatment. The experiments were conducted according to the ethical guidelines of the institutional review board (TSGH‐1‐101‐05‐092, TSGH‐2‐102‐05‐111) of the National Defense Medical Center, Taipei, Taiwan. The histological type of all tumor specimens was T4aN2b, as per World Health Organization criteria.

The oral cancer tissue blocks were implanted subcutaneously into NOD/SCID/IL2R gamma null (NOD.Cg‐Prkdcscid Il2rgtm1Wjl/SzJ; NOD scid gamma) mice (8‐10 weeks), which were maintained in the National Defense Medical Center, Taipei, Taiwan. All experiments were approved by the Institutional Animal Care and Use Committee (14‐299) of the National Defense Medical Center. The tumor growth of xenograft models was monitored at least twice a week. Lengths (longest diameters) and widths (shortest diameters) of the tumors were measured using calipers, and tumor volume was calculated as volume = 1/2 × length × width^2^. If the tumor volume reached approximately 3000 mm^3^, the tumor tissues were removed and sliced into small pieces (approximately 500 mm^3^) for serial transplantation.

When the tumor volume reached approximately 500 mm^3^, mice with seventh‐generation PDTXs (134‐PDTX) were randomized into 2 groups (*n* = 4 per group) receiving TPL (0.15 mg/kg/daily) or phosphate‐buffered saline (PBS; vehicle control) through intraperitoneal injection for 28 days. Body weight and tumor volume were measured at least twice weekly. Tumor size was measured using Vernier calipers twice weekly, and tumor volume was calculated using the aforementioned formula. At the end of the treatment, the mice were sacrificed and the tumors were removed, weighed, and visualized.

### 2.3 Histology and immunohistochemistry (IHC)

TPL‐treated oral cancer SAS xenograft tissues were included in the study.^35^ The mice with 134‐PDTX were sacrificed using CO_2_, and their tissues were fixed through perfusion with 4% paraformaldehyde in 0.1 M phosphate buffer. Next, 5‐μm‐thick serial sections were obtained on slides, deparaffinized in xylene, and rehydrated. After blocking endogenous peroxidase activity using 3% hydrogen peroxide, the slides were incubated with the anti‐DcR3 (333202, BioLegend, San Diego, CA, USA) and anti‐MTA1 (A300‐911A, BETHYL, Montgomery, TX, USA) antibodies overnight at 4°C. Target protein expression was detected using anti‐mouse and anti‐rabbit peroxidase complexes, and peroxidase activity was observed using 3‐amino‐9‐ethyl‐carbazole. The slides were counterstained with hematoxylin (Sigma‐Aldrich) and mounted with a mounting solution.

### 2.4 Evaluation of immunohistochemical staining

The intensity of tumor cells immunoreactivity was scored on a scale of 0‐3 (0, no staining; 1, weak intensity; 2, moderate intensity; and 3, strong intensity). The percentage of tumor cells with nucleus or cytosol staining for each intensity score was graded on a 5‐point scale (0, 0%; 1, 0%‐25%; 2, 25%‐50%; 3, 50%‐75%; and 4, 75%‐100%). Immunostaining scores (range 0‐12) were determined by multiplying the scores based on the percentages of the stained tumor cells (0‐4) with the intensity scores (0‐3). Samples with IHC scores ≥4 were defined as having high DcR3 expression. In the animal studies, immunostaining scores were determined by multiplying the scores based on the percentages of stained tumor cells (0‐4) with the intensity scores (0‐3) and the percentage of survival tumor cells in the tissue.

### 2.5 Cell culture and reagents (cells, siRNA, plasmids, and transfection)

The human tongue squamous cell carcinoma cell line SAS (JCRB0260; JCRB) was provided to us by Dr Lo (Institute of Oral Biology, Department of Dentistry, National Yang‐Ming University, Taipei, Taiwan).^41^ In addition, the tongue cancer cell line SCC25 (CRL‐1628; ATCC) was obtained from the American Type Culture Collection, and HSC‐3 (JCRB0623; JCRB) cells were provided by Dr Lin (Tri‐Service General Hospital, Taipei, Taiwan).^42^ All the tongue squamous cell carcinoma cell lines were cultured in RPMI 1640 media supplemented with 10% fetal bovine serum, 1% penicillin/streptomycin, and 2 mmol/L L‐glutamine. The cells were grown at 37°C in a humidified incubator with a 5% CO_2_ atmosphere.

TPL (Calbiochem, San Diego, California) purity ≥95% as determined using high‐performance liquid chromatography was dissolved in dimethyl sulfoxide to form a 100‐μM stock and then added to cells at the indicated concentrations.

The plasmids expressing shMTA1 were obtained from the RNAi consortium at Academia Sinica. The shMTA1 nucleotide sequences corresponded to MTA1 coding sequences (TRCN0000230496: TGAAGCTGAGAGCAAGTTAAA; TRCN0000230497: TGCGCATCTTGTTGGACATAT; TRCN0000218670: AGACATCACCGACTTGTTAAA). The plasmids expressing MTA1 and DcR3 were obtained from OriGene (Rockville, Maryland). Plasmids were isolated using a GenElute HP EndoFree Plasmid Maxiprep kit (Sigma, St. Louis, Missouri), and transfection was performed using a PolyJet (SignaGen Laboratories Ijamsville, Rockville, Maryland), according to manufacturer instructions.

### 2.6 In vitro cell proliferation assay

Tongue cancer cells (10 000/well in 24‐well plates) were exposed to various concentrations of TPL for 24‐48 hours. Methylene blue assay was used to evaluate the effect of TPL on cell growth, as described previously.^36^

### 2.7 Quantitative real‐time polymerase chain reaction (Q‐PCR)

Total RNA was extracted by using TRIzol Reagent (Invitrogen) according to the manufacturer's protocol. First‐strand cDNA synthesis and amplification was performed using the Maxima H Minus First Strand cDNA Synthesis Kit (Thermo Scientific, Rockford, Illinois). The following Q‐PCR primers were designed using Primer3 (NCBI): DcR3, 5′‐CAGACGTGCAACGACCTGAC‐3′ (forward) and 5′‐TGGGACCTGCATCCTCAC‐3′ (reverse), and GAPDH, 5′‐GGAAGGTGAAGGTCGGAGTCA‐3′ (forward) and 5′‐GTCATTGATGGCAACAATATCCACT‐3′ (reverse). Q‐PCR amplifications were performed using a real‐time PCR system (Applied Biosystems 7500 Fast) using 20‐μL reaction volumes containing 15 μL of SYBR Green PCR Master Mix (Thermo Scientific). Changes in DcR3 expression were calculated using an Applied Biosystems 7500 Real‐Time PCR System (Applied Biosystems 7500 Software, Version 2.0.6).

### 2.8 Western blot analysis

Cell pellets were lysed directly in RIPA buffer containing 50 mM Tris (pH 7.8), 0.15 M NaCl, 5 mM EDTA, 0.5% Triton X‐100, 0.5% NP‐40, 0.1% sodium deoxycholate, a protease inhibitor mixture, and a phosphatase inhibitor mixture (Calbiochem, Billerica, Massachusetts). The protein concentrations of the supernatants were determined using a BCA protein assay kit (Thermo Scientific). For each lane of 10% SDS‐PAGE, 30 μg of cell lysate protein was loaded, separated, and transferred onto a polyvinyldifluoride membrane (GE Healthcare, United Kingdom). The membranes were then probed using specific antibodies against DcR3 (#4758, Cell signaling), MTA1 (A300‐911A, BETHYL), and GAPDH (LF‐PA0018, LabFrontier, Korea).

### 2.9 Statistical analysis

Statistical analyses were performed using GraphPad Prism (GraphPad Software, San Diego, California). Associations between the IHC results and clinicopathological variables were analyzed, using the Chi‐square test. The correlation between DcR3 and MTA1 was assessed by Pearson correlation coefficient test. A Kaplan‐Meier analysis was performed to estimate overall survival, and distributions were compared using the Mantel‐Cox log‐rank test. Differences among studied subgroups were determined using Student's *t* test if normal distributions were evident, and the Mann‐Whitney *U* test was used for nonnormal distributions. *P* < 0.05 was considered as statistically significant.

## 3 RESULTS

### 3.1 DcR3 is a potential biomarker and therapeutic target for human OSCC

**Figure 1:**
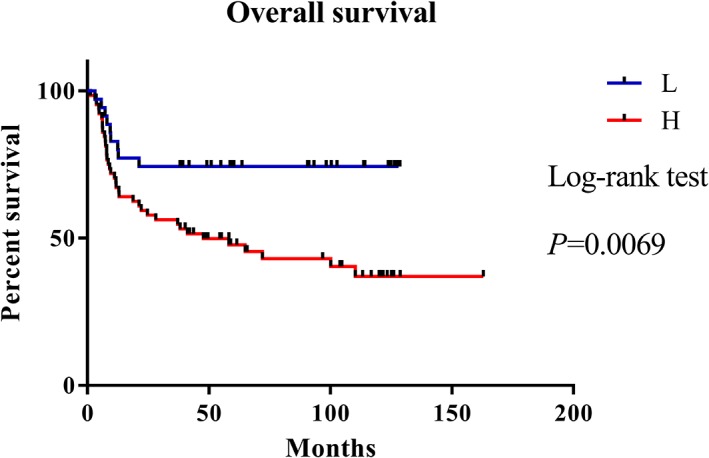
DcR3 is a potential novel diagnostic marker and therapeutic target in oral squamous cell carcinoma (OSCC) patients. Kaplan‐Meier curve compares the overall survival of cancer with high‐level or low‐level DcR3 protein products. Samples with immunohistochemistry (IHC) scores ≥4 were defined as having high DcR3 expression [Color figure can be viewed at wileyonlinelibrary.com]

**Figure 2:**
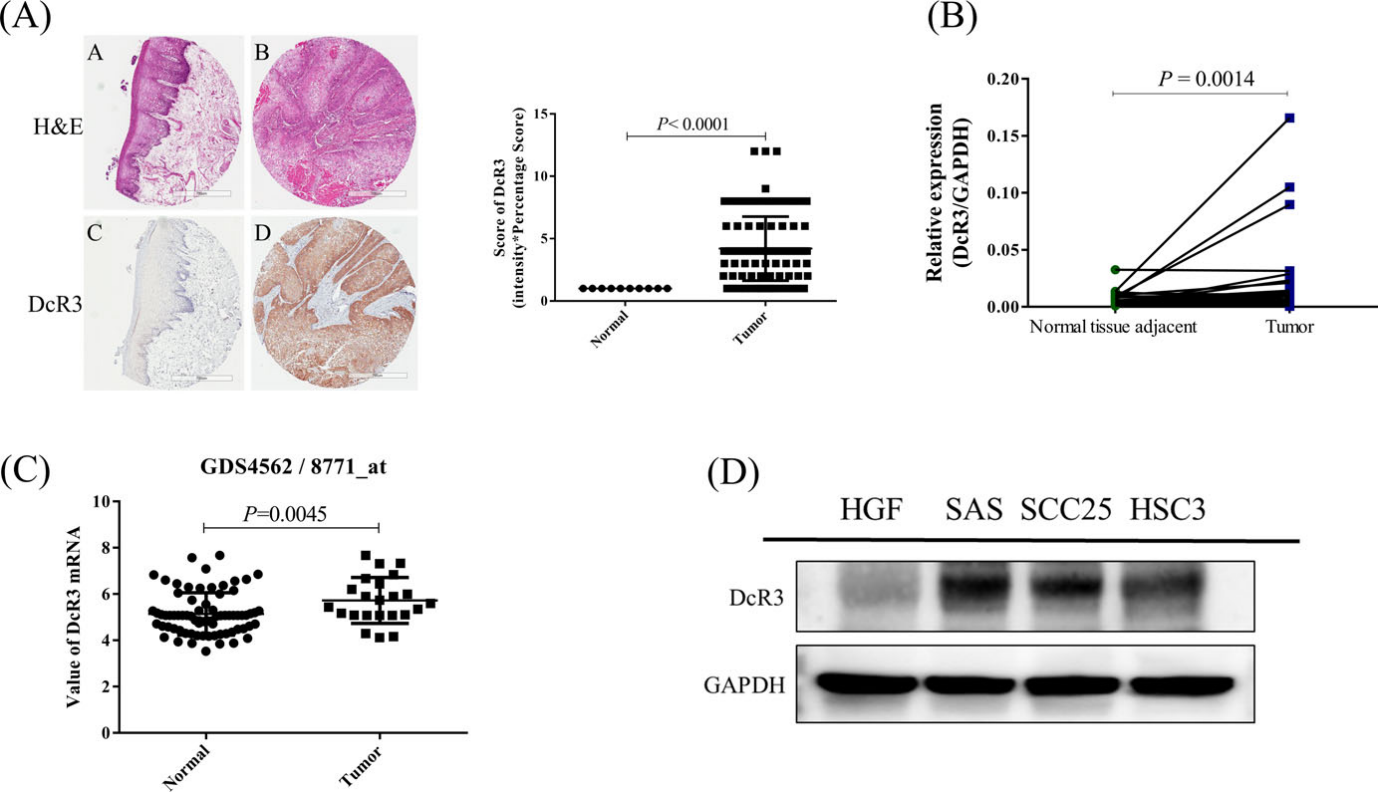
Overexpression of DcR3 in oral cancer. A, Positive cytosol immunostaining of DcR3 in normal mucosa and oral cancer tissues. B, Quantitative polymerase chain reaction results from oral cancer tissues (*n* = 30 patients) and their matched adjacent normal mucosal tissues (*n* = 8 patients). C, DcR3 mRNA expression in human tongue cancer. Data were obtained from NCBI Gene Expression Omnibus profiles (http://www.ncbi.nlm.nih.gov/geoprofiles; Reporter: GDS4562). D, DcR3 protein in three tongue cancer cell lines was determined through Western blot analysis. Normal human gingival fibroblast (HGF) cells were used as negative control. *P* < 0.05 was considered statistically significant [Color figure can be viewed at wileyonlinelibrary.com]

To assess the expression of DcR3 protein in oral cancer cells, we evaluated the status of DcR3 using tissue microarrays of human OSCC cells (*n* = 99; Table 1) containing different oral cancer grades as well as normal mucosal tissues, and the percentage of positive stained cells was calculated as described previously.^43^ DcR3 expression levels were significantly higher in oral cancer tissues than in adjacent normal oral mucosa (*P* < 0.0001; Table 1) and associated with tumor size (*P* = 0.01; Table 1). The Kaplan‐Meier analysis revealed that high staining scores of DcR3 were correlated with poor prognosis (*P =* 0.006; Figure 1). We further analyzed DcR3 mRNA levels in OSCC tissues paired with adjacent normal mucosal tissues from 30 patients; higher DcR3 RNA levels were observed in the OSCC tissues than in the adjacent normal mucosal tissues (*P* = 0.001; Figure 2B). We also used a bioinformatics databank (NCBI Gene Expression Omnibus profiles, GDS4562) to assess the expression of DcR3 in tongue cancer and observed that DcR3 protein levels were higher in OSCC tissues than in normal mucosal tissues (*P* = 0.004; Figure 2C). Western blot analyses of the tongue cancer cell lines (SAS, SCC25, HSC‐3) displayed higher DcR3 expression levels than the normal human gingival fibroblast primary cells (Figure 2D). These results suggest that DcR3 is highly expressed in OSCC, demonstrating its potential as a novel diagnostic marker and therapeutic target.

**Table 1:** Associated between DcR3 expression and multiple clinicopathological parameters in oral squamous cell carcinoma (OSCC)

		DcR3		
Clinicopathological parameters	Cases	Low	High	*P* values
Normal oral mucosa	10	10	0	<0.0001*
OSCC	99	35	64	
Sex				
Male	86	27	59	0.05
Female	13	8	5	
Age				
<52	54	16	38	0.21
≧52	45	19	26	
Tumor size				
T1‐T2	59	27	32	0.01*
T3‐T4	40	8	32	
Cervical node metastasis				
N(−)	49	21	28	0.14
N(+)	50	14	36	
Clinical stage				
I‐II	38	18	20	0.05
III‐IV	61	17	44	
Recurrent				
R(−)	55	22	33	0.29
R(+)	44	13	31	
Location of tumors				
Buccal	38	18	20	
Palate	2	1	1	
Tongue	40	19	21	0.07
Gingiva	13	5	8	
Mouth floor	3	0	3	
Lip	3	2	1	

* mean *P* < 0.05.

### 3.2 TPL inhibited tumor growth in oral cancer PDTX models

**Figure 3:**
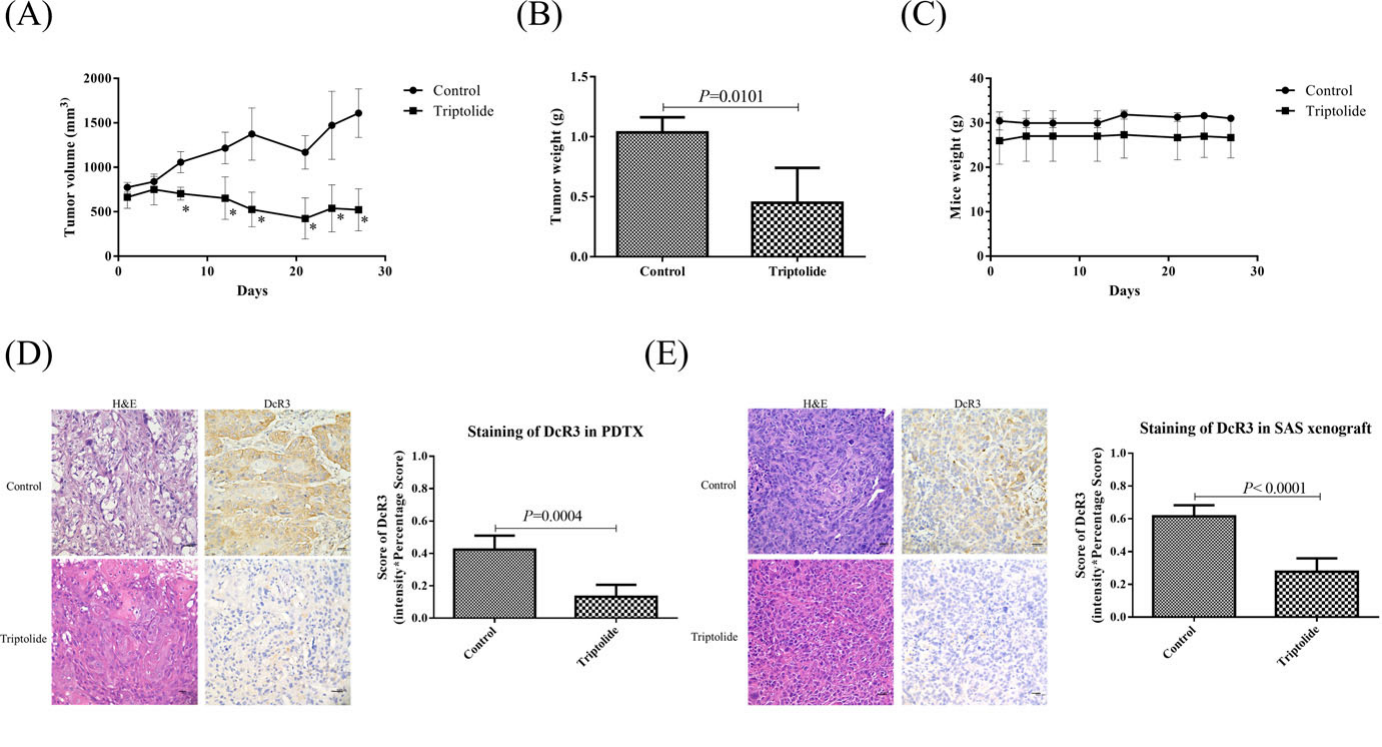
Triptolide (TPL) inhibited tumor growth in DcR3‐overexpressing oral cancer patient‐derived tumor xenograft (PDTX) models. A, Changes in tumor volume in 134‐PDTX models (*n* = 4) treated with TPL (0.15 mg/kg daily intraperitoneally) and phosphate‐buffered saline (PBS) (vehicle control; *n* = 4) for 28 days. Tumor diameters were measured twice weekly for 28 days using Vernier calipers; tumor volume was calculated and compared with those of controls. *P* < 0.05 was considered statistically significant. B, Tumor mass was weighed after the mice were sacrificed. C, No significant change was observed in the body weight of the mice compared with that of the vehicle controls. D, Hematoxylin and eosin staining and immunohistochemistry were performed after administration of TPL or PBS (vehicle control). The 134‐PDTX model stained positive for DcR3. E, The SAS xenograft model stained positive for DcR3. Immunodetectable proteins were stained brown; nuclei were counterstained blue. Original magnification: ×400 [Color figure can be viewed at wileyonlinelibrary.com]

TPL is an effective anticancer compound, but its mechanism of action against oral cancer remains unclear. In the current study, we examined the effects of TPL on growth in the oral cancer patient‐derived PDTX (134‐PDTX) model and found that TPL significantly inhibited tumor growth in the 134‐PDTX model when compared with the vehicle (PBS) controls (Figure 3A, *P* = 0.01; Figure 3B). No apparent toxicity or weight loss was observed after TPL administration during the experimental period (Figure 3C).

### 3.3 TPL repressed DcR3 expression in oral cancer PDTX models and SAS xenografts

IHC analysis verified DcR3 expression in oral cancer cells in the PDTX and SAS xenograft models, and we observed that DcR3 expression was decreased after TPL administrated in clinical tumor tissue‐bearing mice when compared with the controls (Figure 3D). Furthermore, DcR3 expression was significantly decreased in the TPL‐treated groups in both the 134‐PDTX and SAS xenograft models (*P* = 0.0004; Figure 3D, *P* < 0.0001; Figure 3E).

### 3.4 TPL suppressed oral cancer proliferation associated with the DcR3/MTA1 axis

**Figure 4:**
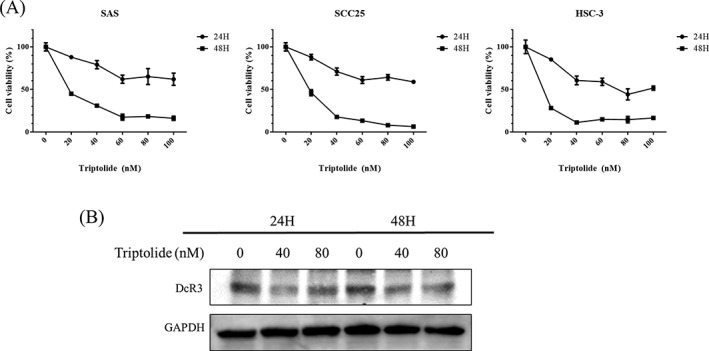
Triptolide (TPL) decreased DcR3 expression in oral cancer. A, Assessment of cell proliferation and viability using the methylene blue assay in the three oral cancer cell lines treated with varying concentrations of TPL (0‐100 nM) or DMSO (1 μL/mL) for 24 and 48 hours. B, Western blot analysis for DcR3 after SAS cells were treated with TPL for 24 and 48 hours

TPL was inhibited the proliferation of oral cancer cells (Figure 4A) and repressed DcR3 expression in a time and dose manner (Figure 4B).

**Figure 5:**
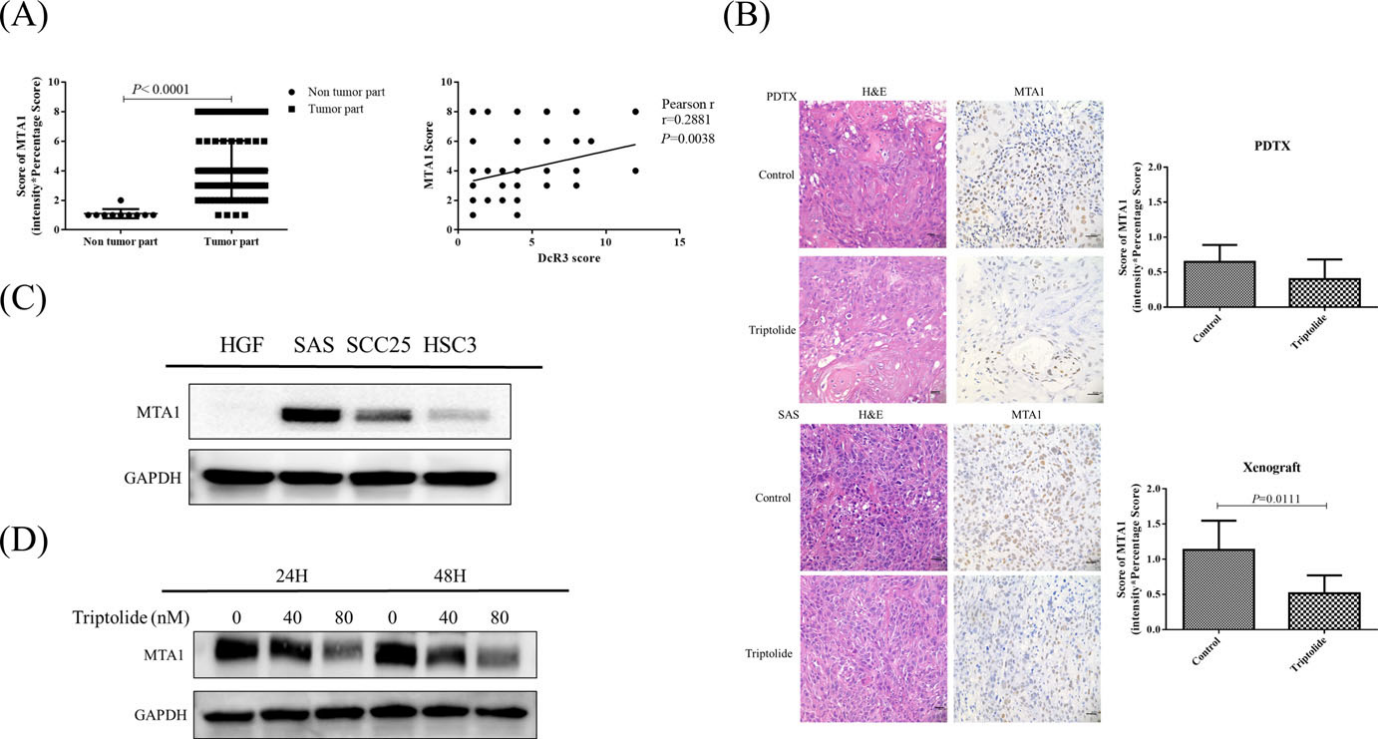
Triptolide (TPL) repressed MTA1 expression in oral cancer. A, Correlation analysis of DcR3 and MTA1 expression in oral squamous cell carcinoma (OSCC) tissue microarray. B, Hematoxylin and eosin staining and immunohistochemistry were performed after administration of TPL or phosphate‐buffered saline (PBS) (vehicle control). The 134‐PDTX and SAS xenograft models stained positive for MTA1. Immunodetectable proteins were stained brown; nuclei were counterstained blue. Original magnification: ×400. C, MTA1 protein in 3 tongue cancer cell lines was determined through Western blot analysis. Normal human gingival fibroblast (HGF) cells were used as negative control. D, Western blot analysis for MTA1 after SAS cells were treated with TPL for 24 and 48 hours [Color figure can be viewed at wileyonlinelibrary.com]

MTA1, a transcription factors of the DcR3 promoter according to the GENECARD transcription factors analysis, was found to be overexpressed in tissues from patients with OSCC in the current study, and it exhibited a positive correlation with DcR3 levels (Pearson *r* = 0.2881; *P =* 0.003; Figure 5A). IHC staining revealed that TPL repressed of MTA1 expression in the PDTX and xenograft tissues (Figure 5B). Furthermore, MTA1 was overexpressed in all 3 OSCC cell lines (Figure 5C); however, TPL was found to suppress its expression in the SAS cell line in a time‐dependent and dose‐dependent manner (Figure 5D).

**Figure 6:**
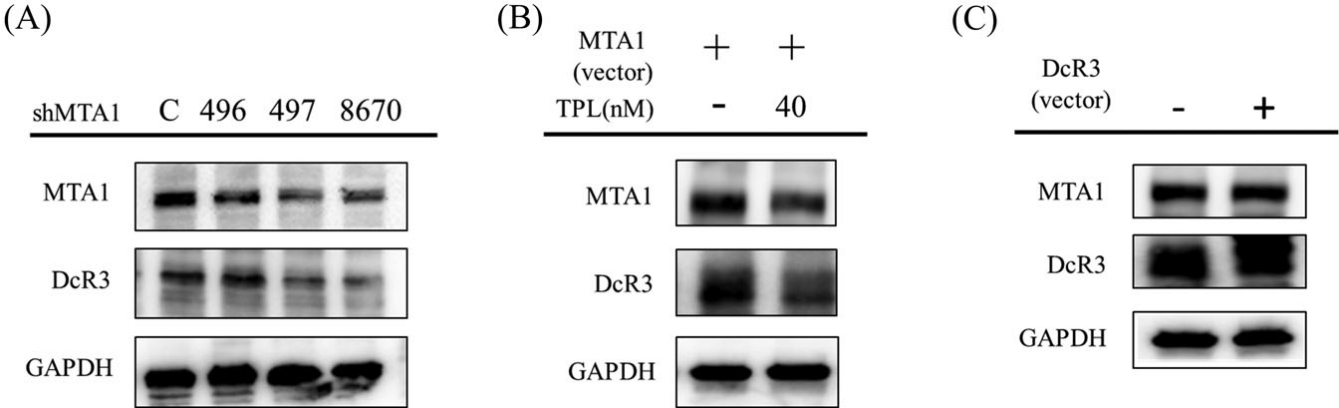
MTA1 regulated DcR3 expression in SAS cancer cells. A, DcR3 expression decreased after introduction of shMTA1 in SAS cells. B, DcR3 was overexpressed after MTA1 was overexpressed, and was subsequently downregulated through triptolide (TPL) treatment in SAS cells. C, Addition of the DcR3‐overexpressed vector was not associated with changes in MTA1 expression in SAS cells

It appeared that MTA1 regulated DcR3 expression in SAS cancer cells (Figure 6). DcR3 expression decreased after introduction of shMTA1 in SAS cells (Figure 6A). DcR3 was overexpressed after MTA1 was overexpressed, and was subsequently downregulated through TPL treatment in SAS cells (Figure 6B). Addition of the DcR3‐overexpressed vector was not associated with changes in MTA1 expression in SAS cells (Figure 6C).

## 4 DISCUSSION

DcR3 expression is upregulated in several inflammatory diseases and malignancies.^4^, ^9^, ^12^, ^17^, ^18^, ^44^, ^45^ Elevated serum level of DcR3 is a potential marker for nodal metastasis of OSCC.^12^ The fact that DcR3 is a secreted molecule makes its detection in the serum by enzyme‐linked immunosorbent assay relatively easy and patient friendly compared with other methods used to diagnose OSCC. Wu et al reported that DcR3 was not detected in tumor‐free patients but was identified in 98.8% (82 of 83) of patients with malignant cancers,^46^ indicating that elevated expression levels of DcR3 are significantly correlated with tumorigenesis and tumor progression. In the present study, we found that high expression levels of DcR3 were correlated with poor survival rates and larger tumor size in OSCC (Table 1 and Figure 1).

PDTX models are progressively used as powerful tools for the preclinical evaluation of anticancer drugs due to their ability to maintain the diversity of molecular histologies and preserve the 3‐dimensional tumor‐stromal cell interactions and components similar to clinical tumor tissues.^47^ Numerous preclinical PDTX models including those of lung cancer,^48^, ^49^, ^50^ breast cancer,^51^ colon cancer,^52^ hepatocellular carcinoma,^53^, ^54^ gastrointestinal stromal tumor,^55^ and melanoma^56^ cells have been established and used for evaluating antitumor compounds;^57^ however, relatively few PDTX models of oral cancer have been developed.^29^ In the present study, NSG mice were used to establish oral cancer PDTX models. The tumor mass was transplanted into NOD‐SCID mice to assess the antioral cancer effect of TPL. According to the IHC analysis, cancer tissues from the PDTX model and the patient were histologically similar. Moreover, TPL could inhibit oral cancer tumor growth and repress the expression of DcR3 (Figure 3) in the PDTX model. In our previous studies, we have demonstrated that TPL also inhibited cell growth in oral cancer xenograft models.^35^, ^36^ Furthermore, a novel compound derived from diterpene triepoxide was demonstrated to reactivate p53 function and significantly decrease tumor progression and volume in vitro, in vivo, and in a PDTX model of human papillomavirus‐positive head and neck squamous cell carcinoma.^58^ Taken together, these results indicated that TPL might be a potential adjuvant drug for OSCC.

TPL, an ancient Chinese herb, has been determined to have significant cytotoxic effects on different types of tumors, including oral cancer.^36^, ^58^ TPL is a diterpenoid epoxide produced by the thunder god vine, *T. wilfordii*, with a molecular weight of 360.4 g/mol, thus belonging to a group of small molecular prodrugs. Consequently, synthetic compounds are being studied in several clinical trials.^59^ Numerous putative target proteins responsible for the antiproliferative activity of TPL have been reported, including HSP70, XBP, and ADAM10^60^, ^61^, ^62^; nevertheless, the anticancer mechanism of TPL remains unclear. Overexpression of DcR3 is thought to promote cancer progression.^4^, ^9^, ^12^, ^17^, ^18^, ^44^, ^45^ TPL has been shown to inhibit tumor growth in pancreatic cancer via the downregulation of DcR3 expression.^39^ In the current study, TPL suppressed tumor growth and repressed the expression of DcR3 in vitro, in vivo, and in PDTX models of OSCC (Figures 3 and 4), suggesting that the antitumor effects of TPL are exerted via repression of DcR3 expression in OSCC.

Aberrant gene expression in cancer is associated with transcription factor activation.^63^, ^64^ By using bioinformatics tools (GENECARD) to scan the transcription factors of DcR3, MTA1 was identified as one of the transcription factors. MTA1 is a component of several chromatin remodeling complexes, including the nucleosome remodeling and deacetylation complex.^65^, ^66^, ^67^ Previous studies revealed that MTA1 is one of the most upregulated proteins in human cancer, and it is associated with cancer progression, aggressive phenotypes, and poor prognosis in patients with cancer.^26^, ^67^ In the present study, both DcR3 and MTA1 were overexpressed in OSCC patients (Figures 2 and 5), and MTA1 was positive correlated with DcR3 expression in the clinical data (Figure 5A). Interestingly, TPL repressed both DcR3 and MTA1 expression in vitro, in vivo, and in the PDTX model of OSCC (Figures 3, 4, 5). These data suggest that TPL is a potential therapeutic option for oral cancers with DcR3 overexpression.

According to bioinformatics studies, MTA1 is a transcription factor of DcR3. TPL can repress the expression of DcR3 and MTA1 in SAS cells. To determine whether the mechanism of TPL's repression of oral cancer was through the DcR3‐MTA1 axis, both the expression and downregulation of the MTA1 vector were applied in SAS cells. We revealed that DcR3 expression was both upregulated and downregulated by the MTA1 vector. However, MTA1 expression was regulated by the DcR3 vector (Figure 6). However, the detailed mechanism of action of TPL in oral cancer requires further investigation.

In summary, this study demonstrated that the anticancer effect of TPL was accompanied by DcR3 downregulation in vitro, in vivo, and in the preclinical PDTX model of oral cancer. Moreover, we posit that DcR3 could be a diagnostic marker and therapeutic target for oral cancer. Furthermore, TPL can potentially be used as an effective chemotherapeutic agent for oral cancer. Finally, this study extends current knowledge by further evaluating the mechanism of action of TPL against oral cancer.

## CONFLICT OF INTEREST

The authors declare that they have no conflicts of interest with the content of this article.
